# Corrigendum: Darbepoetin alfa injection versus epoetin alfa injection for treating anemia of Chinese hemodialysis patients with chronic kidney failure: A randomized, open‐label, parallel‐group, non‐inferiority phase III trail

**DOI:** 10.1002/cdt3.93

**Published:** 2023-09-19

In the article titled, “Darbepoetin alfa injection versus epoetin alfa injection for treating anemia of Chinese hemodialysis patients with chronic kidney failure: A randomized, open‐label, parallel‐group, non‐inferiority Phase III trail” published in pages 59‐70, issue 1, vol. 8 of Chronic Diseases and Translational Medicine,^1^ the information of trial registration at the end of Abstract is missing. The registration information should be: This study has been registered on www.chinadrugtrials.org.cn, registered number CTR20130080.
